# Skywave Detection and Mitigation for the MF R-Mode Continuously Operating Reference Station

**DOI:** 10.3390/s23115046

**Published:** 2023-05-24

**Authors:** Pyo-Woong Son, Jongmin Park, Jaewon Yu, Suhui Jeong, Younghoon Han, Tae Hyun Fang

**Affiliations:** 1 Korea Research Institute of Ships and Ocean Engineering, Daejeon 34103, Republic of Korea; pwson@kriso.re.kr (P.-W.S.); yhhan@kriso.re.kr (Y.H.); 2 Department of Ship and Ocean Engineering Major, University of Science and Technology, Daejeon 34113, Republic of Korea; 3 School of Integrated Technology, Yonsei University, Incheon 21983, Republic of Korea; jm97@yonsei.ac.kr (J.P.); jaewon.yu@yonsei.ac.kr (J.Y.); ssuhui@yonsei.ac.kr (S.J.)

**Keywords:** MF R-Mode, terrestrial navigation, skywave effect, APNT, positioning accuracy

## Abstract

There is an increasing need for an independent terrestrial navigation system, owing to the increasing reliance on global navigation satellite systems (GNSS). The medium-frequency range (MF R-Mode) system is considered a promising alternative; however, the skywave effect caused by ionospheric changes at night can degrade its positioning accuracy. To address this problem, we developed an algorithm to detect and mitigate the skywave effect on MF R-Mode signals. The proposed algorithm was tested using data collected from Continuously Operating Reference Stations (CORS) monitoring the MF R-Mode signals. The skywave detection algorithm is based on the signal-to-noise ratio (SNR) induced by the groundwave and skywave composition, whereas the skywave mitigation algorithm was derived from the I and Q components of the signals obtained through IQ modulation. The results demonstrate a significant improvement in the precision and standard deviation of the range estimation using CW1 and CW2 signals. The standard deviations decreased from 39.01 and 39.28 m to 7.94 and 9.12 m, respectively, while the precision (2-sigma) increased from 92.12 and 79.82 m to 15.62 and 17.84 m, respectively. These findings confirm that the proposed algorithms can enhance the accuracy and reliability of MF R-Mode systems.

## 1. Introduction

The global navigation satellite system (GNSS) is a vital component of the modern world, enabling precise positioning and navigation for various applications such as transportation, mapping, and military operations [1,2,3]. GNSS is based on a constellation of satellites that orbit the Earth and transmit signals received by GNSS receivers on Earth. These signals are then used to calculate the position, velocity, and time of the receiver. GNSS can be used in navigation systems for aviation, shipping, and other modes of transportation, as well as in financial systems, communication networks, and energy grids [4]. The widespread reliance on GNSS has made it an important component of modern society, and its availability and accuracy are essential for ensuring the safety, efficiency, and security of critical systems and applications.

However, GNSSs are vulnerable to various types of interference, such as jamming and spoofing, which can compromise their accuracy and reliability, resulting in significant consequences for GNSS users [5,6]. For example, GNSS is critical for ensuring the safety and efficiency of vehicles in the transportation sector, particularly in autonomous systems such as self-driving cars. In the event of GNSS interference, these vehicles can become unguided, leading to potential accidents. Considering these security concerns, multiplexing the navigation system using a radio navigation system on the ground can ensure safe navigation for GNSS users [7].

Terrestrial radionavigation systems such as the enhanced long-range navigation (eLoran) system and the medium-frequency (MF) R-Mode system can provide a backup to the GNSS to mitigate its vulnerabilities [8,9,10]. These systems use ground-based radio signals to provide navigation information to users, offering a highly accurate and reliable alternative to GNSS [11]. Unlike GNSS signals, which are susceptible to jamming and interference, terrestrial navigation signals are much less vulnerable to such threats and can provide navigation information in areas where GNSS signals are weak or unavailable. Therefore, the deployment of terrestrial navigation signals can increase the resilience of navigation systems and ensure the safety and efficiency of navigation, particularly in critical applications such as aviation, shipping, and national defense [12].

Terrestrial radionavigation signals vary in propagation velocity depending on the characteristics of the propagation environment, causing changes in the time of flight of the navigation signals and becoming the main error source in position estimation [13,14]. This variation is influenced by regional and temporal factors such as terrain, weather, and season. To compensate for this, it is necessary to continuously monitor signals in the service area and generate correction information. The eLoran system can provide position estimation with an accuracy of approximately 10 to 20 m by applying real-time correction information [15]. This is similar to the concept of Continuously Operating Reference Stations (CORS) operated by the GNSS [16].

The skywave effect is a phenomenon that occurs when the D layer of the ionosphere disappears as the sun sets, causing eLoran or MF R-Mode signals to be refracted back to the surface of the Earth [17,18]. This effect can cause a significant degradation in the accuracy of terrestrial radionavigation systems, considering that the travel time of the navigation signals is affected by changes in the refractive index of the atmosphere [19]. The eLoran system is designed to be resistant to the skywave effect by using pulsed signals; however, the MF R-Mode system, which uses continuous wave signals, is susceptible to the skywave effect, thereby decreasing the performance of the system. Several MF R-Mode tests conducted in the Baltic Sea showed that the accuracy of a ship’s positioning is significantly reduced at night, owing to the impact of the skywave effect [20].

South Korea conducted a research project to develop MF R-Mode technology that could be used in case of GNSS failure by implementing four MF R-Mode transmission stations at Socheong-do (SC), Palmi-do (PM), Chungju (CJ), and Eocheong-do (EC), and one CORS to achieve a 10 m position accuracy in the nearby seas. The MF R-Mode CORS monitors changed across a broad region and generated correction information. However, the skywave effect can be difficult to predict as it occurs suddenly and irregularly in different areas of the ionosphere, making it difficult to consistently apply to users over a large area. Therefore, the impact of the skywave effect must be excluded to generate accurate correction information for the MF R-Mode CORS.

This paper presents an algorithm to eliminate the skywave effect that occurs at night, and verifies its performance. First, we describe the construction of the CORS for the MF R-Mode system in detail and analyze the phase change of the MF R-Mode signal according to the time variation at the CORS. Subsequently, a signal-to-noise ratio (SNR)-based skywave detection method and an IQ modulation-based skywave reduction algorithm are proposed. Finally, the verification of the effectiveness of the algorithm through actual experiments near the CORS is presented in detail.

## 2. Continuously Operating Reference Station of the Korean MF R-Mode System

The area near Daesan Port, which serves as the testbed in this study for validating the performance of the MF R-Mode, has various propagation paths with different distances and terrains to each of the four MF R-Mode transmission stations: SC, PM, CJ, and EC. In general, the range estimation accuracy for MF R-Mode signals tends to decrease with an increase in the propagation path length, owing to signal strength attenuation. However, this attenuation is relatively less pronounced when the signal travels over sea, compared to on land. Specifically, for an MF R-Mode signal with the same power amplitude, range estimation can be performed with higher precision when the distance to the user receiver is shorter and the propagation path has a lower percentage of land.

**Figure 1:**
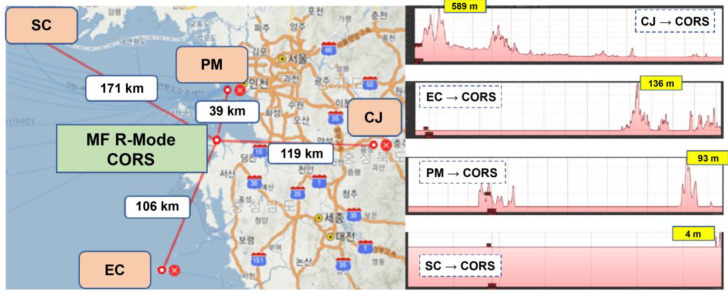
Geometric distribution of the four MF R-Mode transmitters and the lengths of the propagation paths (**left**), and elevation profiles of each propagation path acquired from Google Earth (**right**). The yellow box in each profile presents the highest altitude along the propagation path.

The propagation paths of signals from the SC and PM transmission stations were the longest and shortest, with approximate lengths of 171 and 39 km, respectively, mostly over the sea. The signal from the CJ transmission station, which had a propagation path length of approximately 119 km, was mostly over land and propagated through mountainous areas near the transmission station. The signal from the EC transmission station had the characteristic of passing through both land and sea, to some extent. The map on the left-hand side of Figure 1 shows the geometric arrangement of the four MF R-Mode transmitters and the Daesan CORS and the distances of the signals. The waveforms on the right-hand side show the elevation profiles of the signal propagation paths from each transmitter to the MF R-Mode CORS, which were produced from Google Earth. 

### 2.1. Horizontal Dilution of Precision

The position dilution of precision (PDOP) is a term used in satellite navigation systems, such as the Global Positioning System (GPS), to describe the geometric quality of the satellite configuration as seen from a receiver [21]. The PDOP values provide information about the relative accuracy of the GPS position solution given the current satellite geometry. The smaller the PDOP value, the better the satellite geometry and the more accurate the positioning solution. Furthermore, the concept of PDOP can be applied to terrestrial-based navigation systems. A low PDOP between the transmitter and user implies that a higher accuracy in position estimation can be achieved when receiving the same signal quality. Consequently, analyzing the positioning accuracy performance based on the PDOP of CORS can help to predict the positioning accuracy performance that users can use in other marine regions.

Generally, the PDOP includes the distribution of satellites and receivers along the *x*-, *y*-, and *z*-axes based on the Earth-centered, Earth-fixed (ECEF) coordinate system. However, the propagation system based on terrestrial signals is different from the satellite navigation system, and only calculates the horizontal position based on the World Geodetic System 1984 (WGS84) coordinate system; therefore, the horizontal dilution of precision (*HDOP*) was used. The *HDOP* can be derived as follows:

(1) $$A=[\begin{array}{ccc} \sin\theta_{1} & \cos\theta_{1} & 1\\ \sin\theta_{2} & \cos\theta_{2} & 1\\ \vdots & \vdots & \vdots \\ \sin\theta_{n} & \cos\theta_{n} & 1 \end{array}],$$

(2) $$Q={\left(A^{T}A\right)}^{-1},$$

(3) $$HDOP=\sqrt{Q{\left(1,1\right)}^{2}+Q{\left(2,2\right)}^{2}},$$

where $\theta_{i}$ indicates the azimuth angle between the CORS position and the *i*-th MF R-Mode transmitter.

### 2.2. Equipment Installation for Data Collection

**Figure 2:**
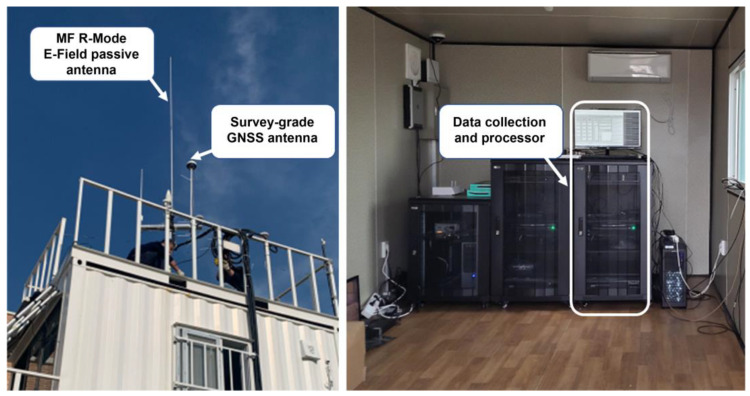
Installation of equipment at CORS to collect and monitor MF R-Mode signals. MF R-Mode E-field passive antenna and survey-grade GNSS antenna on the rooftop (**left**), Serco’s MF R-Mode receiver (MFR-1a), and display devices in the shelter (**right**).

In the MF R-Mode CORS testbed, facilities were assembled to continuously receive and monitor MF R-Mode signals to generate correction information that compensates for the phase change of MF R-Mode signals changing over time. To achieve this, MF R-Mode and GNSS antennas were installed on the roofs of the CORS facilities, as shown in Figure 2, and Serco’s MF R-Mode receiver (Model: MFR-1a) was used to install the signal-collection and processing device. The receiver from Serco is the only commercially available MF R-Mode receiver whose performance has been verified through a Complementary Positioning, Navigation, and Timing (PNT) performance validation conducted by the U.S. Department of Transportation [22].

The MFR-1a specification features a marine beacon band front end, designed to enhance the signal by removing noise and interference from adjacent low-frequency (LF) and amplitude-modulated (AM) bands. This front end has a gain range of 0–20 dB and amplifies the signal in the frequency range of 285–325 kHz. The signal is then processed using an analog-to-digital converter (ADC) that records the entire marine beacon band as a single signal for synchronous processing. The ADC operates with a 10 MHz clock signal from an external rubidium clock and has a sample rate of 1 MHz, with 16-bit resolution for a wide dynamic range. Additionally, the MFR-1a specification includes a separate GNSS receiver, which provides a position for initial receiver calibration and ensures the long-term stability of the rubidium clock.

### 2.3. Diurnal Phase Variation at the MF R-Mode CORS

The MF R-Mode signals are produced by updating the differential global navigation satellite system (DGNSS) infrastructure, which transmits GNSS correction information. The signals had a bandwidth of 100 or 200 bps and were transmitted using minimum shift key (MSK) modulation centered at a frequency of approximately 300 kHz. To generate the MF R-Mode signal, a continuous wave (CW) was inserted into the null point in the spectrum of the MSK signal. In Europe, where the signal bandwidth is 100 bps, a null was created 225 Hz away from the center frequency. Meanwhile, in South Korea, where the signal bandwidth is 200 bps, a CW signal was added 250 Hz away from the center frequency.

The phenomenon of diurnal phase changes in LF or MF signals refers to the changes in the phases of these signals over the day owing to the effective conductivity of the surface and humidity of the atmosphere. In eLoran systems using LF signals, the time of arrival (TOA) changes over time; therefore, real-time correction information is generated to compensate for the changes in TOA based on the differential eLoran stations [9].

**Figure 3:**
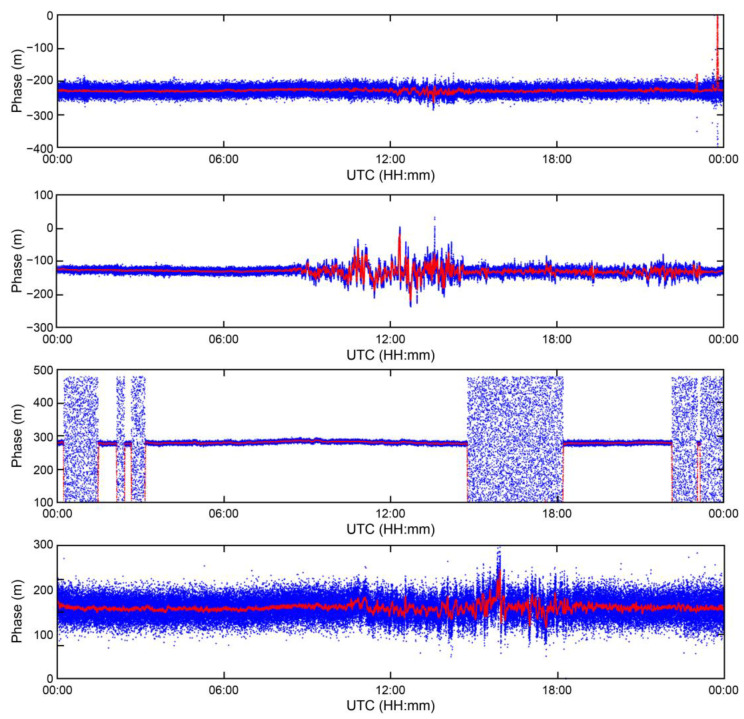
Phase variation of MF R-Mode signals in meters during a 24 h campaign at CORS. The blue dots indicate the raw measurements from the receiver, and red dots indicate smoothed signals using a 100 s moving average filter.

Figure 3 illustrates the results of converting the phases of the signals of the four MF R-Mode transmitters into units of meters over a 24 h period on 8 January 2023. The phases of the MF R-Mode signals ranged between 0 and 2 pi, and could be converted into a range within the wavelength range corresponding to the frequency of the signal, from 0 m. The blue dots represent the phase of the MF R-Mode signal output by the receiver, while the red dots correspond to the result of applying a 100-s moving average function to remove noise and observe changes over time.

The phases of the four MF R-Mode signals exhibit 30 m changes over the course of the day, and real-time correction information must be generated from CORS to compensate for the daily changes to achieve the project goal of a 10 m positioning accuracy with a 95% probability. Some of the transmitters located on the island face challenges in transmitting stable MF R-Mode signals owing to power issues. Consequently, the phases of the received signals had random values at certain times, as shown in the first and third figures of Figure 3. Therefore, CORS should alert users to avoid using these signals for position estimation.

Furthermore, one of the most significant observations from the 24 h MF R-Mode signal data was that the phase underwent large fluctuations between 09:00 and 23:00 UTC. Considering that sunrise and sunset in Korea occurred at 23:32 and 08:43 UTC, respectively, on that day, this was likely because of skywave interference. In addition, the intensity of skywave interference may differ depending on factors such as the distance to each transmission station and the environment of signal propagation; however, it seems necessary to reduce skywave influence to attain a positioning accuracy of 10 m.

### 2.4. Skywave Effect at the MF R-Mode CORS

The skywave effect of MF signals refers to the reflection of MF radio waves from the ionosphere back into the Earth. As the sun sets and the Earth moves into darkness, the D layer of the ionosphere disappears, increasing the height of the ionosphere. This indicates that MF signals can no longer be absorbed by the D layer and, instead, are reflected back to Earth by the higher E layer of the ionosphere [23]. This allows MF signals to travel over the horizon and reach greater distances, making the skywave effect more effective for nighttime MF signals in terms of long-distance communication.

In the MF R-Mode system, which calculates the distance through variations in the phases of signals, the presence of the skywave effect can create a new composite signal along with the ground wave that travels along the surface path. Consequently, the receiver receives erroneous measurements in terms of signal strength and phase, which may take the form of fading signals or multipath. Ultimately, the skywave effect has a detrimental impact on the positioning accuracy from a navigation system standpoint, rather than from a communication perspective.

**Figure 4:**
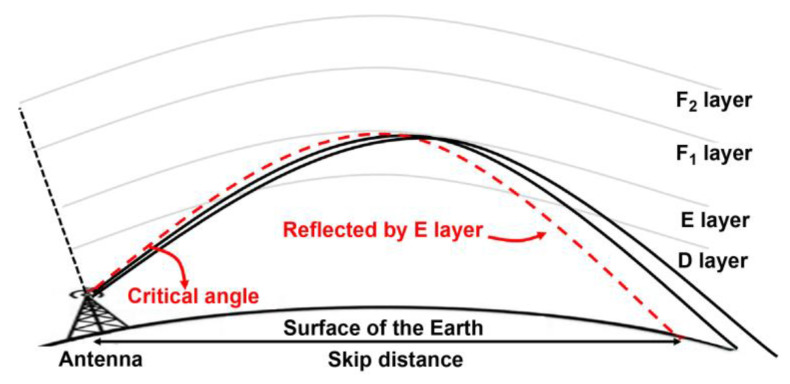
Illustration of the skywave propagation phenomenon and its key parameters, including critical angle, skip distance, and ionosphere.

Even if the D layer of the ionosphere vanishes at night, reflected signals cannot be received in all regions owing to the skywave effect. It is possible to receive reflected signals from the ionosphere only from distances greater than the skip distance. The skip distance refers to the maximum distance that a radio wave can travel along the Earth’s surface before being reflected back to Earth by the ionosphere. In contrast, the critical angle is the maximum angle at which a radio wave must be transmitted for it to be reflected back to Earth by the ionosphere. As shown in Figure 4 and Equation (4), the skip distance ($d_{s}$) is determined by the critical angle ($\theta_{c}$) and the height of the E layer of the ionosphere (h).

(4) $$d_{s}=\frac{2h}{\tan\theta_{c}}$$

Previous studies on the impact of skywaves on MF signals show that the skip distance is 45 km from the transmitter [18], indicating that the positioning accuracy may be impacted at night in areas further than 45 km from the MF R-Mode signal transmitter. Recent experimental results indicate the absence of skywave effects at locations approximately 60 km from the transmitter [19]. However, there has also been evidence that skywave effects can be observed within an approximate range of 50 km, depending on the presence of abrupt mountainous regions along the transmission path [24].

A prior study [18] suggested that we can compare the signal attenuations experienced when MF signals travel 60 km and 500 km through paths consisting solely of land surface and solely of ocean surface, respectively, and verify the attenuations to be of the same degree. The composition of the propagation path, and not just the distance from the transmitter, can result in varying levels of amplitude attenuation. Consequently, it is more appropriate to assess the presence of skywave effects by considering the change in SNR and the variability of the groundwave reduction based on the features of the propagation path, rather than simply relying on the distance from the transmitter.

## 3. Skywave Detection and Mitigation Algorithm

### 3.1. SNR-Based Skywave Detection Algorithm

Assuming the received signal at the receiver (S) is composed solely of groundwaves and skywaves, without considering various other noises, it can be expressed as follows:

(5) $$S\left(t\right)=A_{g}\left(t\right)\cos\left(2\pi f_{c}t+\theta_{g}\left(t\right)\right)+A_{s}\left(t\right)\cos(2\pi f_{c}t+\theta_{s}\left(t\right)),$$

where $A_{g}\left(t\right)$ denotes the amplitude of the groundwave, $f_{c}$ denotes the center frequency of the MF R-Mode signal, $\theta_{g}\left(t\right)$ represents the phase according to the propagation path of the groundwave, $A_{s}\left(t\right)$ indicates the amplitude of the skywave, and $\theta_{s}\left(t\right)$ represents the phase according to the propagation path of the one-hop skywave.

During the daytime, most skywaves are absorbed by the D layer of the ionosphere; therefore, $A_{s}\left(t\right)$ can be assumed to converge to 0. As a result, $A_{g}\left(t\right)$ is nearly the same as the amplitude of S(t), and hence it would represent a stable value as long as the environment along the propagation path does not rapidly change. However, during nighttime, when the skywave reaches the surface, the ratio of $A_{g}\left(t\right)$ to $A_{s}\left(t\right)$ becomes significant and cannot be ignored.

Serco’s MF R-Mode receiver, which is installed at the MF R-Mode CORS, computes the SNR by comparing the magnitude of the center frequency of the MF R-Mode signal to that of the surrounding frequencies, with no signal in the fast Fourier transform (FFT) spectrum of the received data over a 1-s interval. This enables fluctuations in the signal strength to be reflected in the SNR of the MF R-Mode signal, providing a means to detect the presence of the skywave effect. Such multipath detection technology utilizing the detection of SNR changes is already used in fields such as GNSS-R [25].

**Figure 5:**
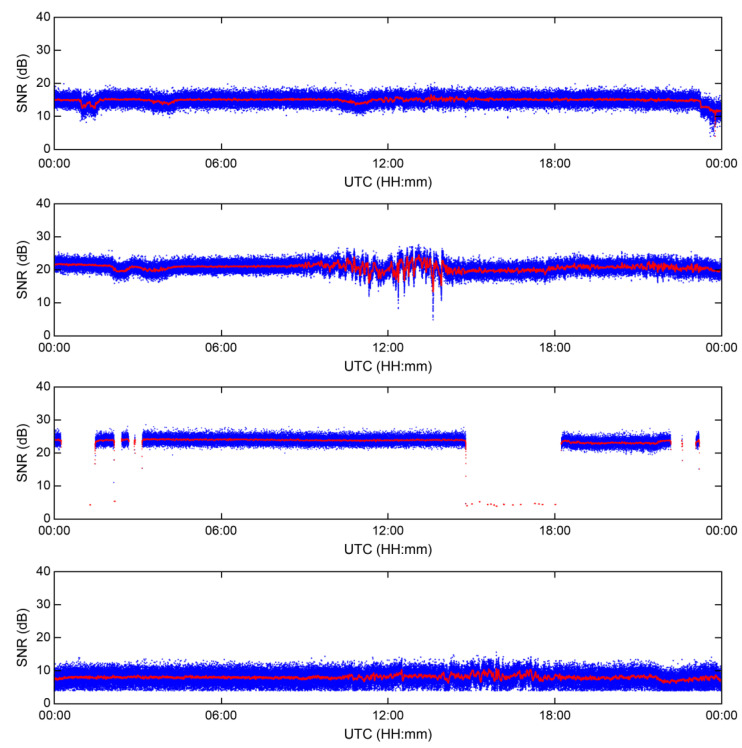
SNR variation of MF R-Mode signals in meters received from EC, CJ, PM, and SC transmitters in sequence during the 24 h campaign at CORS. The blue dots indicate the raw measurements from the receiver, and red dots indicate smoothed signals using a 100 s moving average filter.

Figure 5 shows the SNRs received from each transmitter, i.e., EC, CJ, PM, and SC, over 24 h. The data were collected over the same durations as those shown in Figure 3. The blue and red dots indicate the SNR output from the receiver and the results after passing through a moving average filter for 100 s, respectively.

The proposed skywave detection algorithm entails the following steps. The output SNR from the receiver is processed through a 100 s moving average filter (represented by the red points in Figure 5). If the standard deviation of these results, calculated over a 100 s time period, exceeds the standard deviation mean value of the 100 s moving average SNR over the course of the day, the skywave effect takes place as per Equation (6). The threshold for this detection and the length of the moving average filter were established experimentally using long-term data collected at the CORS.

(6) $$skywave\;flag=1\;if\;\sigma_{\bar{SNR}}\left(t\right)>\bar{\sigma_{\bar{SNR}}}$$

**Figure 6:**
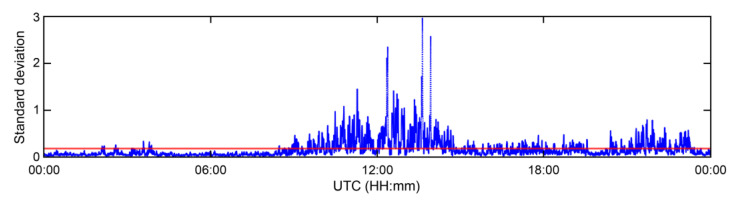
Variation in standard deviation of MF R-Mode signal’s SNR from CJ transmitter (denoted in blue) and the threshold of determining the presence of skywave (denoted in red).

For example, the proposed algorithm was applied to the MF R-Mode signal from the CJ transmitter, which was expected to be the most affected by skywaves among the signals received at CORS, considering the distance and topographical features of the propagation path from the transmitter. In Figure 6, the statistical values for detecting the skywave effect are represented by blue dots, and the threshold is represented by a red line. The time when the statistical values exceeded the threshold was almost similar to the time when the phase changed abruptly, as shown in Figure 4.

### 3.2. Skywave Mitigation Algorithm Using IQ Modulation

In IQ modulation, the amplitude and phase of a carrier signal are modulated using two separate signals, referred to as in-phase (*I*) and quadrature (*Q*) signals. The *I* and *Q* signals are typically generated by mixing the carrier signal with two separate local oscillator signals, one of which is shifted in-phase by 90°. The *I* and *Q* signals can be expressed mathematically as

(7) $$\begin{array}{ll} \begin{array}{c} I(t)=S(t)\cos(2\pi f_{c}t)\\ Q(t)=-S(t)\sin(2\pi f_{c}t) \end{array} & . \end{array}$$

Here, $f_{c}$ is the carrier frequency, and I(t) and Q(t) are the in-phase and quadrature signals, respectively. The in-phase signal controls the amplitude of the carrier signal, whereas the quadrature signal controls the phase of the carrier signal.

The received signal can be separated into its *I* and *Q* components by using IQ modulation, enabling us to analyze and manipulate each component independently. This is particularly useful in situations where there are multiple paths for the signal to reach the receiver, resulting in multipath interference and signal fading. By analyzing the *I* and *Q* components independently, we can detect and correct for the effects of multipath interference, leading to a more accurate and reliable signal.

The demodulation process using the phase angle of the complex baseband signal in IQ demodulation involves computing the arctangent of the ratio of quadrature and in-phase signals. This results in a phase-angle value that represents the phase of the original carrier signal. Typically, a complex baseband signal is represented as a complex number, with in-phase and quadrature signals forming the real and imaginary components of the complex number, respectively. The phase angle (θ) of the complex baseband signal can be computed as

(8) $$\theta =\arctan(\frac{Q(t)}{I(t)}).$$

**Figure 7:**
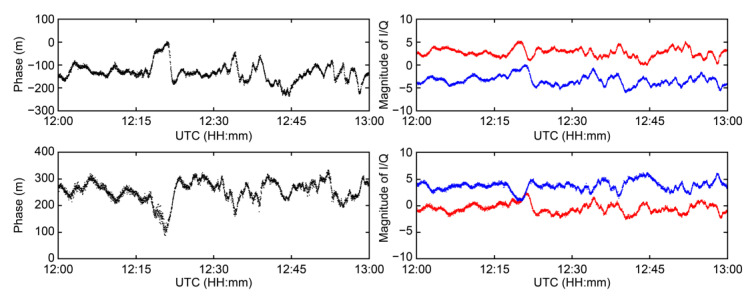
Analysis of the phase and I/Q signals of the MF R-Mode signal from the CJ transmitter during a 1 h campaign at CORS: CW1 (**upper**) and CW2 (**bottom**) comparison.

MFR-1a, a Serco MF R-Mode receiver, estimates the range of the signal by utilizing the phase angle calculated from the *I* and *Q* components of the complex baseband of the MF R-Mode signal, which is subsequently converted from radians to meters. MFR-1a can simultaneously receive up to five signals from MF R-Mode transmitters and output the calculated *I* and *Q* values each second. Figure 7 shows the phase and I/Q of the MF R-Mode signal from the CJ transmitter within an hour, with significant phase changes owing to the skywave effect within 24 h. The MF R-Mode signal comprises two CWs that are shifted by the same frequency difference from the center frequency in the positive and negative directions. In this study, signals with frequencies higher than the center frequency were defined as CW1, whereas those with frequencies lower than the center frequency were defined as CW2. The first row of Figure 7 shows the CW1 signal, and the second row shows the CW2 signal.

When observing the phase graphs in column 1 of Figure 7, it can be observed that both CW1 and CW2 experience significant phase changes of several hundred meters for 1 h owing to the skywave effect. Furthermore, the trends in these changes exhibit opposite directionality. However, this correlation was found to be more pronounced when the IQ graph in column 2 of Figure 7 was examined. The red and blue lines in the IQ graph represent the *I* and *Q* components, respectively. Furthermore, the *I* component of CW1 (the red line in the upper-right graph) exhibits a completely opposite trend to that of the *Q* component of CW2 (the blue line in the lower-right graph). In addition, the *Q* component of CW1 (blue line in the upper-right graph) exhibits a trend similar to that of the *I* component of CW2 (red line in the lower-right graph).

Based on long-term data collected at CORS, the aforementioned characteristics have been consistently observed without loss of generality. Based on these observations, we established two assumptions regarding the characteristics that occur when the skywave effect is present.

**Assumption** **1.** *The variation in the I component of CW1 is negatively correlated with the variation in the Q component of CW2.*

**Assumption** **2.** *The variation in the Q component of CW1 is positively correlated with the variation in the I component of CW2.*

An algorithm to implement these two assumptions can be expressed as follows:

(9) $${\hat{\theta }}_{\mathrm{CW}1}=\arctan^{-1}(\frac{Q_{\mathrm{CW}1}-\frac{\Delta Q_{\mathrm{CW}1}+\Delta I_{\mathrm{CW}2}}{2}}{I_{\mathrm{CW}1}-\frac{\Delta I_{\mathrm{CW}1}-\Delta Q_{\mathrm{CW}2}}{2}}),$$

(10) $${\hat{\theta }}_{\mathrm{CW}2}=\arctan^{-1}(\frac{Q_{\mathrm{CW}2}-\frac{\Delta Q_{\mathrm{CW}2}-\Delta I_{\mathrm{CW}1}}{2}}{I_{\mathrm{CW}2}-\frac{\Delta I_{\mathrm{CW}2}+\Delta Q_{\mathrm{CW}1}}{2}}),$$

where ${\hat{\theta }}_{\mathrm{CW}1}$ and ${\hat{\theta }}_{\mathrm{CW}2}$ denote the phases of the two MF R-Mode signals with mitigated skywave effects, respectively.

## 4. Results

**Figure 8:**
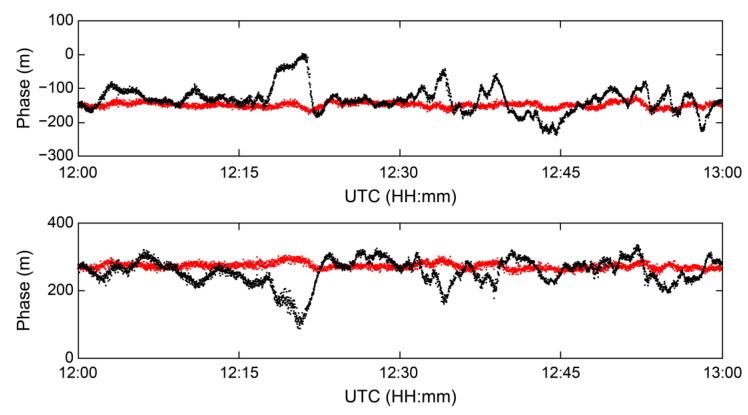
Improvement in the mitigating skywave effect at CORS by applying the proposed algorithm. The black and red dots show the raw measurements from the receiver and the result of the proposed algorithm, respectively.

The proposed algorithm was applied to the MF R-Mode signal from the CJ transmitter, which is most affected by skywaves at the CORS in Daesan Port, to verify its performance. Figure 8 shows the phases of the CW1 and CW2 signals from the MRF-1a receiver (black line), and the phases estimated again after applying the proposed algorithm (red line) provided by the receiver.

The measured phase values for the CW1 and CW2 signals exhibited standard deviations of 39.01 and 39.28 m, respectively, with a 95% level of precision, yielding values of 92.12 and 79.82 m, respectively. However, on applying the proposed algorithm, the standard deviations of the CW1 and CW2 signals were reduced to 7.94 and 9.12 m, respectively, whereas the 95% levels of precision were improved to 15.62 and 17.84 m, respectively. The results are presented in Table 1.

**Figure 9:**
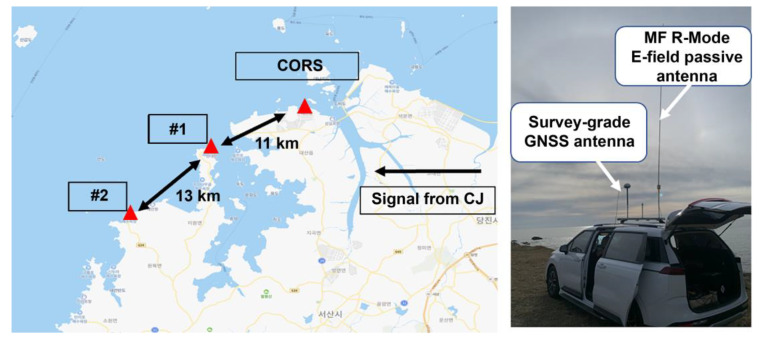
Geometric distribution of CORS and two additional experiment sites (**left**), and potable experimental setting on the vehicle (**right**).

To confirm whether the proposed algorithm can be applied regardless of the receiver location, additional experiments were conducted at two points near the CORS. An antenna park was set up to install GNSS and MF R-Mode antennas on the vehicle loop, and the same signal-collection setup as in the CORS was implemented using an MFR-1a receiver. As shown in Figure 9, the two additional experimental locations were approximately 11 and 24 km away from the CORS and were selected at clear coastal locations to minimize the influence of the terrain.

The distances between the new measurement points (#1, #2) and the CJ transmitter were approximately 10 and 20 km longer, respectively, than the distance between the CORS and CJ transmitter; however, precision degradation did not increase proportionally with the distance, as shown in Table 2, owing to the complex terrain in Korea. Based on the additional validation at these two locations, we can confirm that our proposed algorithm can significantly reduce the impact of skywaves and improve precision. Notably, the effect of error reduction caused by the skywave in the second additional site is remarkable, with a reduction of 10 to 20 times.

## 5. Conclusions

We developed an algorithm to detect and mitigate the skywave effect of MF R-Mode signals caused by ionospheric changes at night, and evaluated its performance. The MF R-Mode system, a terrestrial navigation system that functions as an independent navigation system when GNSS is used in difficult circumstances in the ocean, uses signals in the medium-frequency band. However, the signal is negatively affected by the skywave reflected from the E layer when the ionospheric D layer disappears at night, which significantly decreases the system’s accuracy. 

We installed CORS as a testbed to detect and minimize the skywave effect, and then collected signals from 2021 for our investigation. Because a skywave is a type of multipath signal, it combines with a groundwave that propagates through the ground, causing a rapid change in SNR. Therefore, we proposed a skywave detection algorithm using the SNR. Furthermore, because ionospheric changes are very irregular and unpredictable, it is difficult to secure the ground truth regarding the occurrence of skywaves. However, we confirmed that we could detect the moments when the phase changed significantly using the change in the SNR.

Additionally, by observing the data for a long period of time, we confirmed that we can effectively remove skywaves by using the *I* and *Q* signals generated in IQ modulation, and verified the performance of the algorithm using the data from the hour when the skywave effect was most severe. Furthermore, we confirmed that the proposed algorithm can be applied regardless of the receiver’s location, through experiments conducted at two additional locations along with the data from CORS. The algorithm proposed in this study can utilize signals that otherwise could not be used for position determination owing to the skywave effect, and this is expected to have a positive impact on the availability and accuracy performance of the MF R-Mode system.

## Figures and Tables

**Table 1:** Statistics about the improvement in the mitigation of the skywave effect at CORS.

	Receiver Output		Proposed Algorithm Applied	
	CW1	CW2	CW1	CW2
Standard Deviation	39.01 m	39.28 m	7.94 m	9.12 m
Precision (95%)	92.12 m	79.82 m	15.62 m	17.84 m

**Table 2:** Statistics about the improvement in the mitigation of the skywave effect at additional two sites.

Standard Deviation	Receiver Output		Proposed Algorithm Applied	
	CW1	CW2	CW1	CW2
#1	25.63 m	20.78 m	9.57 m	13.78 m
#2	130.49 m	61.74 m	4.97 m	6.93 m
**Precision (95%)**	**Receiver Output**		**Proposed Algorithm Applied**	
	**CW1**	**CW2**	**CW1**	**CW2**
#1	46.73 m	41.36 m	18.17 m	25.43 m
#2	253.99 m	125.39 m	9.82 m	14.56 m

## Data Availability

Not applicable.
